# Non-Specific Blocking of miR-17-5p Guide Strand in Triple Negative Breast Cancer Cells by Amplifying Passenger Strand Activity

**DOI:** 10.1371/journal.pone.0142574

**Published:** 2015-12-02

**Authors:** Yuan-Yuan Jin, Jade Andrade, Eric Wickstrom

**Affiliations:** 1 Biochemistry & Molecular Biology, Thomas Jefferson University, Philadelphia, Pennsylvania, United States of America; 2 Chemistry, Haverford College, Haverford, Pennsylvania, United States of America; 3 Sidney Kimmel Cancer Center, Thomas Jefferson University, Philadelphia, Pennsylvania, United States of America; Ben-Gurion University, ISRAEL

## Abstract

Conventional wisdom holds that only one of the two strands in a micro ribonucleic acid (miRNA) precursor duplex is selected as the active miRNA guide strand. The complementary miRNA passenger strand, however, is thought to be inactive. High levels of the oncogenic miRNA (oncomiR) guide strand called miR-17-5p is overexpressed in triple negative breast cancer (TNBC) and can inhibit ribosomal translation of tumor suppressor gene mRNAs, such as programmed cell death 4 (*PDCD4*) or phosphatase and tensin homolog (*PTEN*). We hypothesized that knocking down the oncogenic microRNA (oncomiR) miR-17-5p might restore the expression levels of PDCD4 and PTEN tumor suppressor proteins, illustrating a route to oligonucleotide therapy of TNBC. Contrary to conventional wisdom, antisense knockdown of oncomiR miR-17-5p guide strand reduced PDCD4 and PTEN proteins by 1.8±0.3 fold in human TNBC cells instead of raising them. Bioinformatics analysis and folding energy calculations revealed that mRNA targets of miR-17-5p guide strand, such as *PDCD4* and *PTEN*, could also be regulated by miR-17-3p passenger strand. Due to high sequence homology between the antisense molecules and miR-17-3p passenger strand, as well as the excess binding sites for the passenger strand on the 3’UTR of *PDCD4* and *PTEN* mRNAs, introducing a miR-17-3p DNA-LNA mimic to knock down miR-17-5p reduced PDCD4 and PTEN protein expression instead of raising them. Our results imply that therapeutic antisense sequences against miRNAs should be designed to target the miRNA strand with the greatest number of putative binding sites in the target mRNAs, while minimizing affinity for the minor strand.

## Introduction

New cases of aggressive breast cancer are predicted to occur in 232,340 U.S. women in 2014, and to kill 39,620 [1]. Triple-negative breast cancers (TNBC) lack estrogen receptor (ER), progesterone receptor (PR), and human epidermal growth factor receptor 2 (ErbB2, Her2), and represent 16% of cases (2). Without specific molecular targets, conventional chemotherapy of TNBC yields modest clinical outcomes [2]. Thus, the 16% of breast cancer patients who suffer from TNBC have worse prognoses than other subtypes of breast cancer [3] [2].

TNBC clearly needs new molecular therapies that specifically target genes promoting cancer cell survival. A variety of oncogenic micro ribonucleic acids (oncomiRs) are overexpressed in TNBC, and are being studied intensively as targets for complementary oligonucleotide therapy [4]. OncomiRs are non-protein-coding RNAs of 18–25 nucleotides (nt) that form base pairs with specific sequences in mRNAs. They inhibit translation of mRNAs sterically or by inducing mRNA degradation by Ago2 [5, 6]. Biogenesis of all miRNAs initiates in the nucleus, where primary miRNAs are transcribed by either RNA polymerase II or RNA polymerase III. Primary miRNA transcripts are then processed by Drosha and its cofactor DGCR8 to produce shorter precursor miRNA hairpins of ~70 nt [7]. Pre-miRNA hairpins are exported to the cytoplasm by exportin 5, then cleaved by Dicer to yield double-stranded miRNAs. The guide strand of the double-stranded miRNA is thought to exhibit weak hydrogen bonding at its 5’ end, favoring its binding to Ago2 in an RNA-induced silencing complex (RISC), allowing the guide strand to be active against complementary mRNAs. The passenger strand is thought to be inactive, dissociated, and degraded [8].

Specific oncomiR target recognition is predominantly defined by Watson-Crick base pairing that occurs between the seed region (nucleotide 2 to 8 from the 5’ end of the oncomiR guide strand) and the 3’-untranslated regions (3’UTR) of target mRNAs. Translational repression by oncomiRs can be achieved by perfect complementarity between oncomiRs and the 3’UTR of mRNAs mediated by RISC, leading to mRNA degradation. Alternately, the translation of mRNA is sterically inhibited through imperfect oncomiR-mRNA recognition [9]. On average, each oncomiR has hundreds of possible mRNA targets [10]. As a result, complementary oligonucleotide therapy against one oncomiR could impact a broad panel of genes.

The miR-17~92 cluster is one of the most studied of the oncomiR groups that play important roles in cancer development. miRNAs from this cluster are generally up-regulated in various cancers, including breast, lung, colon, pancreas, prostate, and gastric cancer [11, 12]. Caloric restriction (CR) and ionizing radiation (IR) down-regulate members of the miR-17~92 cluster in TNBC models, decreasing their metastatic activities by suppressing extracellular matrix (ECM) mRNAs that exhibit miR-17-5p binding sites [13]. Among the seven members of the miR-17~92 cluster, the guide strand miR-17-5p is predominantly responsible for promoting migration and invasion of metastatic cancer cells, targeting the mRNAs of tumor suppressor genes, such as *PDCD4* (programmed cell death 4) and *PTEN* (phosphatase and tensin homolog) [14]. Thus, miR-17-5p is considered to be an oncogenic miRNA, or oncomiR. The tumor suppressor proteins PDCD4 and PTEN are usually depressed in TNBC, associated with elevated oncomiR levels [15–19].

The oncomiR miR-17-5p is significantly up-regulated in mesenchymal MDA-MB-231 TNBC cells compared to the noninvasive luminal MCF7 cells, and contributes to the invasiveness and migratory behavior of TNBC [20]. Most TNBCs are basal-like [21, 22], and transcription profiling has suggested that most basal-like TNBC cells have molecular properties of mesenchymal tumors [2]. Using MDA-MB-231 cells as a mesenchymal TNBC cell model, we hypothesized that knocking down miR-17-5p might restore the expression levels of PDCD4 and PTEN tumor suppressor proteins, illustrating a route to oligonucleotide therapy of TNBC.

## Materials and Methods

### OncomiR Target Prediction

Three different oncomiR target prediction algorithms, rna22, TargetScan, and Miranda [23–26] were used to search for oncomiRs that might target *PDCD4* and *PTEN* mRNAs. These 3 databases were chosen because of the diverse algorithms they employ to find targets. rna22 uses non-canonical seed pairing, which allows mismatches in the miRNA seed:mRNA interaction. It utilizes pattern discovery to find matching sequence patterns from a miRNA:mRNA set, then rank predicted miRNA:mRNA pairs based on free energy calculations [27]. rna22 allows G-U wobble base pairs and mismatches in the seed region. Miranda is also less stringent about seed matching by allowing G-U wobble base pairs. Although it considers matching sequences along the entire miRNA:mRNA pair, the final prediction favors matching in the seed region. TargetScan relies heavily on conservation of 3’UTR interactions, followed by fully complementary seed regions that are conserved. It does not allow mismatches or G-U wobble base pairs, although it does consider 3’ compensatory regions.

### Cell Line and Cell Culture

The human mesenchymal TNBC cell line MDA-MB-231 was obtained from American Type Culture Collection (ATCC). MDA-MB-231 cells were characterized as basal B subtype TNBC, with mesenchymal features that allow them to migrate readily and degrades their ability to adhere and polarize [28]. MDA-MB-231 cells were maintained in L-15 medium (ATCC) containing 10% fetal bovine serum (FBS) and 100 U/mL of penicillin/streptomycin (Invitrogen) in a humidified incubator at 37°C without added CO_2_.

### Knockdown Oligonucleotides

Antisense DNA-LNA chimeras were acquired from Exiqon to knock down miR17-5p (5’-dACCTGCACTGTAAGCACTTTG-3’), miR17-3p (5’-dTACAAGTGCCTTCACTGCAG-3’), and miR-21-5p (5’-dCAACATCAGTCTGATAAGCT-3’). Comparison of calculated pure DNA masses with manufacturer’s reported masses for anti-miR-17-5p (MW = 6619.3 Da), anti-miR-17-3p (MW = 6287.1 Da), and anti-miR-21 (MW = 6337.2 Da), implied ~7 LNA residues, probably at the 3’ terminus.

### Mfold Energy Calculation and Structural Prediction of oncomiR:mRNA duplexes

Gibbs free energies (folding energy ΔG°s) were calculated using Mfold [29] at http://mfold.rna.albany.edu/?q=mfold. Since Mfold only allows a single sequence input, a linker sequence (5’-GCGGGGACGC-3’) was inserted between each oncomiR:mRNA pair [30].

### Molecular Dynamics Structural Prediction of oncomiR:mRNA duplexes

Theoretical structures of duplexes were simulated in explicit water, 100 mM NaCl, pH 7.0, at 300°K, with Amber 12 using the ff99SB force field [31, 32] as before [33, 34].

### Antisense DNA-LNA treatment

1.5×10^5^ MDA-MB-231 cells were seeded in 6-well plates in complete medium without antibiotics the day before transfection. Antisense DNA-LNA chimeras (Exiqon) were transfected into MDA-MB-231 cells with 5 μg Lipofectamine 2000 (Invitrogen) at a final oligonucleotide concentration of 50 nM for 6 hours at 37°C in Opti-MEM (Invitrogen) under 5% CO_2_, according to the manufacturer’s protocol. At the end of transfection, cells were washed, then incubated in complete growth medium for another 12 to 48 hours before harvesting.

### miRNA mimic treatment

1.5×10^5^ MDA-MB-231 cells were seeded in 6-well plates in complete medium without antibiotics the day before transfection. Either miR-17-5p mimic or miR-17-3p mimic (Life Technologies) were transfected into MDA-MB-231 cells with 5 μg Lipofectamine 2000 (Invitrogen) at a final oligonucleotide concentration of 50 nM for 6 hours at 37°C in Opti-MEM (Invitrogen) under 5% CO_2_, according to the manufacturer’s protocol. At the end of transfection, cells were washed, then incubated in complete growth medium for another 48 hours before harvesting.

### Real-Time Quantitative PCR

Total RNA from MDA-MB-231 cells was extracted using a mirVana miRNA isolation kit (Life Technologies) according to the manufacturer’s protocol. For qPCR of miRNAs, 10 ng of purified total RNA were reverse transcribed with a TaqMan miRNA reverse transcription kit (#4366597, Life Technologies). qPCR of miRNAs was performed with a miRNA Gene Expression Assay (Life Technologies) on a 7500 Fast Real-Time PCR System (Life Technologies). Primers specific for miR-17 (Assay ID 002308), miR-21 (Assay ID 000397) and internal control RNA U6 (Assay ID 001973) for both reverse transcription and qPCR were obtained from Applied Biosystems. The average absolute values of triplicate samples for the same miRNA were calculated and normalized to U6 RNA, measured by the comparative Ct (2^-ΔΔCt^) method [35].

For qPCR of tumor suppressor mRNAs, 500 ng of purified total RNA were reverse transcribed with a High Capacity cDNA Reverse Transcription Kit (Life Technologies, cat#4368814). qPCR of mRNAs was performed using a FastStart Essential DNA Green Master (Roche, cat#06402712001) on a LightCycler^®^ 96 system (Roche) with the following primers to detect transcripts: *GAPDH* forward primer (5’-dTCCCTCCAAAATCAAGTGGGG-3’), *GAPDH* reverse primer (5’-dGCAAATGAGCCCCAGCCTTC-3’); *PDCD4* forward primer (5’-dGGGAAGGTTGCTGGATAGGC-3’), *PDCD4* reverse primer (5’-dCTCCTGCACCACCTTTCTTTG-3’); *PTEN* forward primer (5’-dGGACCAGAGACAAAAAGGGAGT-3’), *PTEN* reverse primer (5’-dCCAGATGATTCTTTAACAGGTAGC-3’). The average of triplicate samples for the same mRNA was calculated and normalized to the internal control gene *GAPDH*, by the comparative Ct (2^-ΔΔCt^) method [35].

### Western Blots

Cells were trypsinized and harvested with 1×PBS, then lysed in cell lysis buffer (Invitrogen) with protease inhibitor cocktail (P-2714, Sigma). Lysate protein concentrations were quantified by the Bradford Assay (Bio-Rad). Lysate aliquots containing 30 μg protein were separated on NuPAGE 4–12% Bis-Tris gels (Invitrogen), transferred to PVDF membranes, blocked with blocking buffer, and incubated with antibodies against PDCD4 protein (ab80590, Abcam), PTEN protein (9552S, Cell Signaling), and β-actin (AM4302, Ambion), followed by incubation with secondary antibodies labeled with horseradish peroxidase (Invitrogen). The resulting protein bands were imaged by luminescence using a SuperSignal West Femto Chemiluminescent Substrate (Thermo Scientific), on a Kodak Image Station 2000R, and analyzed with Molecular Imaging Software version 5.0.2.30 (Carestream).

### 3’UTR construct cloning

Luciferase constructs containing all predicted binding sites for miR-17-5p or miR-17-3p from the 3’UTRs of *PDCD4* or *PTEN* mRNAs were constructed using a pMir-Report luciferase reporter vector (AM5795, Ambion). To create the luciferase constructs containing the entire 3’UTR of *PDCD4s* mRNA, we PCR amplified the fragment from genomic DNA of MDA-MB-231 cells using forward primer (5’–GCAACTAGTAAGCGAAGGAGATGGAGGTC– 3’), reverse primer (5’–AAACGTTGCCCAAACGAGAGCAAT– 3’), and Phusion High-Fidelity PCR Kit (F-553S, Thermo Scientific) following the manufacturer’s protocol. The amplified PCR product was inserted into the pMir-Report luciferase vector between the SpeI and PmeI restriction sites.

To generate luciferase constructs containing individual predicted 3’UTR binding sites, we purchased the DNA sequence of each binding site, along with its flanking sequence region, as sense and antisense primers (Fisher Scientific). The primers were then annealed and cloned into the pMir-Report luciferase vector between the SpeI and HindIII restriction sites. The sequence of each primer used to generate 3’UTR fragments for cloning is shown in S1 Fig.

### Luciferase assay

8×10^4^ MDA-MB-231 cells were seeded in 24-well plates in complete medium without antibiotics the day before transfection. Cells were transfected with 400 ng of each of the reporter constructs, 100 ng of pRL-TK *Renilla* luciferase internal control vector (E2241, Promega), and with either miR-17-5p mimic or miR-17-3p mimic using Lipofectamine 2000 (Invitrogen, CA). In parallel, transfections without the mimics were performed. After 24 hours, cells were lysed and 50 μL of each lysate was transferred to 96-well plates. Dual-Glo reporter assays were performed according to the manufacturer’s protocol (E2920, Promega). Luminescence intensity for each sample was measured with a Veritas Microplate Luminometer (Turner BioSystems), and each value from pMir-Report firefly luciferase was normalized by pRK-TK *Renilla* luciferase.

### Statistical Analysis

All experimental measurements were performed independently at least three times. Significance was assessed by Student’s t-test.

## Results

### *PDCD4* mRNA is a potential target for miR-17-5p

**Fig 1:**
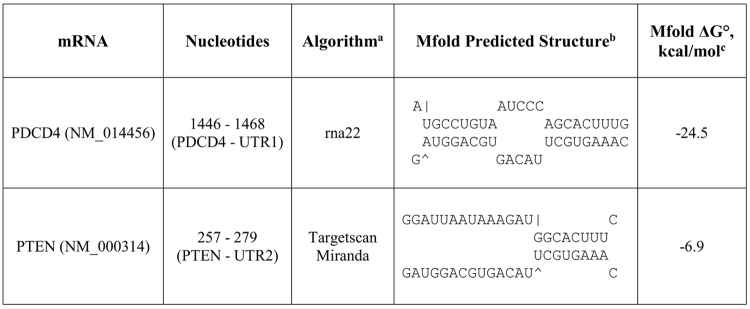
Predicted miR-17-5p guide strand binding sites in the 3'UTR of *PDCD4* and *PTEN* mRNAs. ^a^The potential oncomiR:mRNA binding sites were identified by rna22, Targetscan, or miRanda. ^b^Top strand is mRNA (5' ➔ 3') and the bottom strand is oncomiR (3' ➔ 5'). ^c^Calculated at http://mfold.rna.albany.edu/?q=mfold.

Using rna22, TargetScan and miRanda, we searched for oncomiR targets in *PDCD4* and *PTEN* mRNAs. rna22 identified miR-17-5p as a potential gene regulator through its interaction with one binding site in the 3’UTR of *PDCD4* mRNA (Fig 1). Although rna22 is the only algorithm that predicted a binding site for miR-17-5p in the 3’UTR of *PDCD4* mRNA, the predicted 23 bp oncomiR:mRNA duplex is stable, containing 17 complementary basepairs and an Mfold predicted folding energy ΔG° of -24.5 kcal/mol at 37°C (Fig 1).

### DNA-LNA chimeras knocked down oncomiRs. However, PDCD4 and PTEN protein levels were unexpectedly decreased

**Fig 2:**
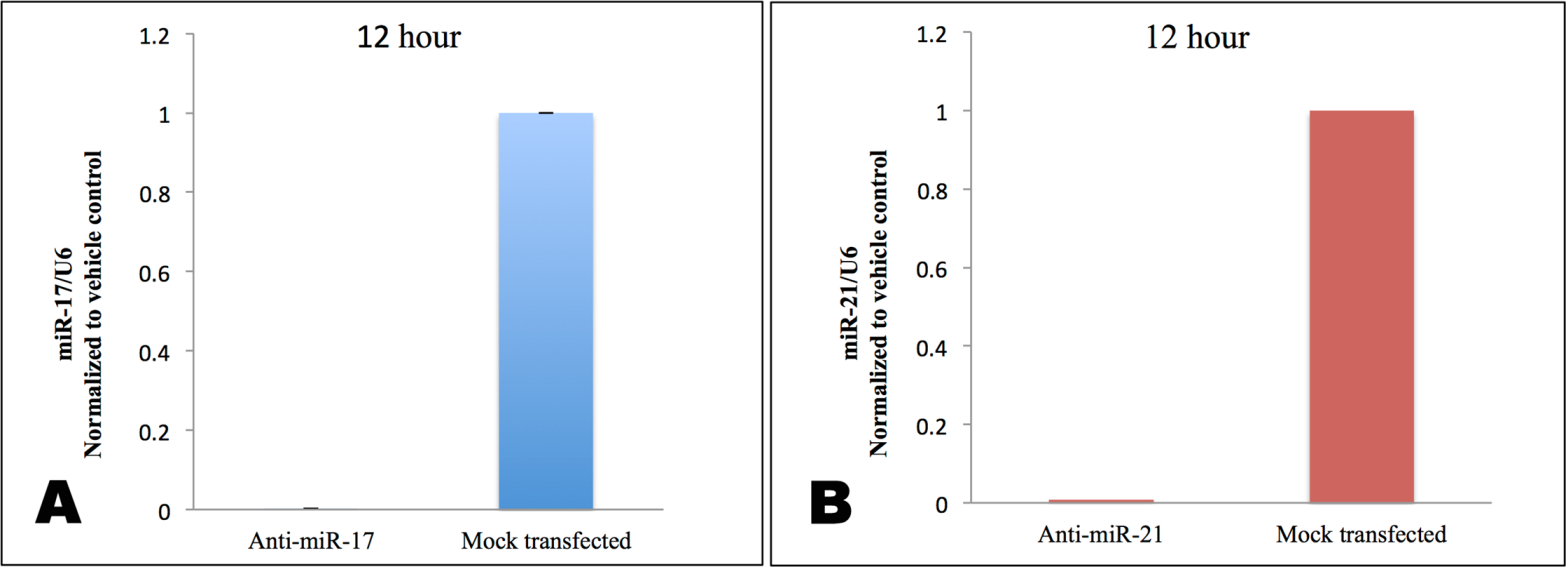
Treatment with complementary DNA-LNA chimeras knocked down endogenous miRNAs in MDA-MB-231 TNBC cells. **A**: qPCR of miR-17-5p 12 hr and 48 hr post-transfection with anti-miR-17-5p. **B**: qPCR of miR-21-5p 12 hr and 48 hr post-transfection with anti-miR-21-5p. Results represent absolute values of miRNA/internal control U6 normalized to mock transfected. Values are the average of three measurements ±.

**Fig 3:**
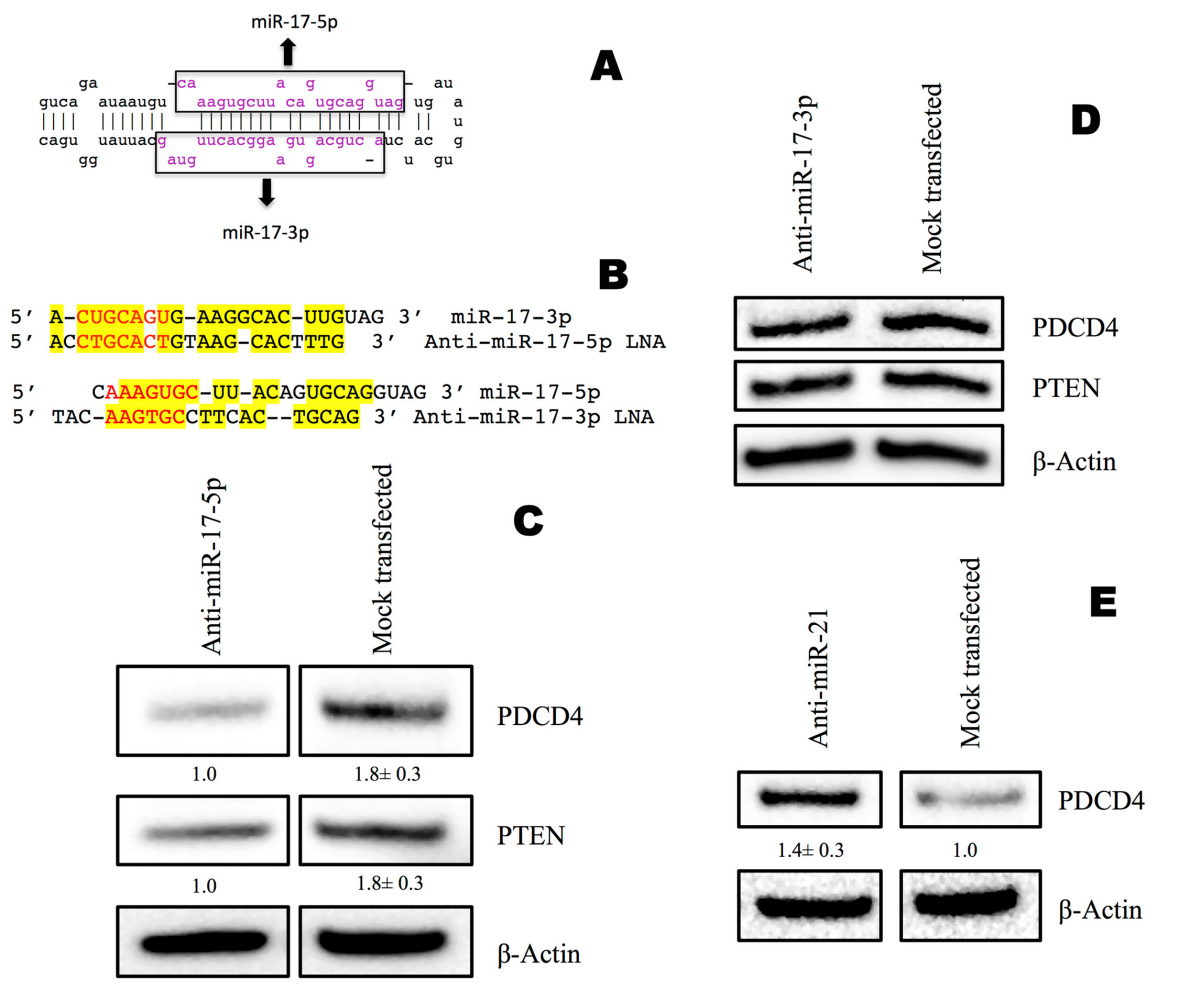
miR-17-3p is a potential regulator of PDCD4 protein level and competes with miR-17-5p for inhibition of *PDCD4* and *PTEN* mRNAs. miR-21-5p guide strand regulates PDCD4 protein level without competing with its passenger strand miR-21-3p. **A**: Mirbase search of miR-17-3p, forming the lower arm of the miR-17 pre-miRNA hairpin. **B**: Homologous sequences between miR-17-5p and miR-17-3p are highlighted in yellow. **C**: PDCD4 and PTEN protein Western blots at 48 hr post transfection with anti-miR-17-5p. **D**: PDCD4 and PTEN protein Western blots at 48 hr post transfection with anti-miR-17-3p. **E**: PDCD4 protein Western blot at 48 hr post transfection with anti-miR-21. β-actin was used as loading control. Values are the average of three blots ± s.d. after normalization to β-actin and to control/treatment group. Each blot was subjected to gamma setting adjustments.

To elucidate the effect of miR-17-5p on *PDCD4* or *PTEN*, endogenous miR-17-5p was knocked down using a commercially available DNA-LNA inhibitor. Anti-miR-17-5p DNA-LNA chimera transfected into MDA-MB-231 TNBC cells knocked down miR-17-5p by 99±0.01% after 12 hr (Fig 2A). To evaluate whether miR-17-5p had any effect on the expression level of PDCD4 protein, we analyzed protein levels 48 hr after transfection. Surprisingly, the PDCD4 protein level was down-regulated by 1.8±0.3 fold, instead of being up-regulated as expected following miR-17-5p knockdown (Fig 3C).

To determine whether the complementary DNA-LNA chimeras were working as predicted, we examined another direct target of miR-17-5p, *PTEN* mRNA [14]. Both miRanda and TargetScan predicted one binding site for miR-17-5p in the 3’UTR of *PTEN* mRNA (Fig 1). Surprisingly, we found that knocking down miR-17-5p decreased PTEN protein level by 1.8±0.3 fold (Fig 3C). This result conflicted with the expectation that miR-17-5p target expression would increase when miR-17-5p was knocked down. These results implied that mature miR-17-5p is a gene regulator of *PDCD4* and *PTEN* mRNA translation. However, anti-miR-17-5p resulted in down-regulation of both *PDCD4* and *PTEN* mRNA translation, rather than stimulation.

### miR-17-3p passenger strand is a potential inhibitor of *PDCD4* and *PTEN* mRNAs, as well as miR-17-5p

To understand the unexpected results in Fig 3B, we examined miR-17 in miRBase. In the pre-miRNA, miR-17-5p was predicted to hybridize with its passenger strand miR-17-3p to form a hairpin (Fig 3A). Most of the miR-17-3p is fully complementary to its guide strand miR-17-5p. Since anti-miR-17-5p is fully complementary to miR-17-5p, its sequence is highly homologous to miR-17-3p (Fig 3B). Therefore, we speculated that anti-miR-17-5p DNA-LNA chimera could act as a miR-17-3p mimic, binding to miR-17-3p target sites in the 3’UTR of *PDCD4* and *PTEN* mRNAs.

**Fig 4:**
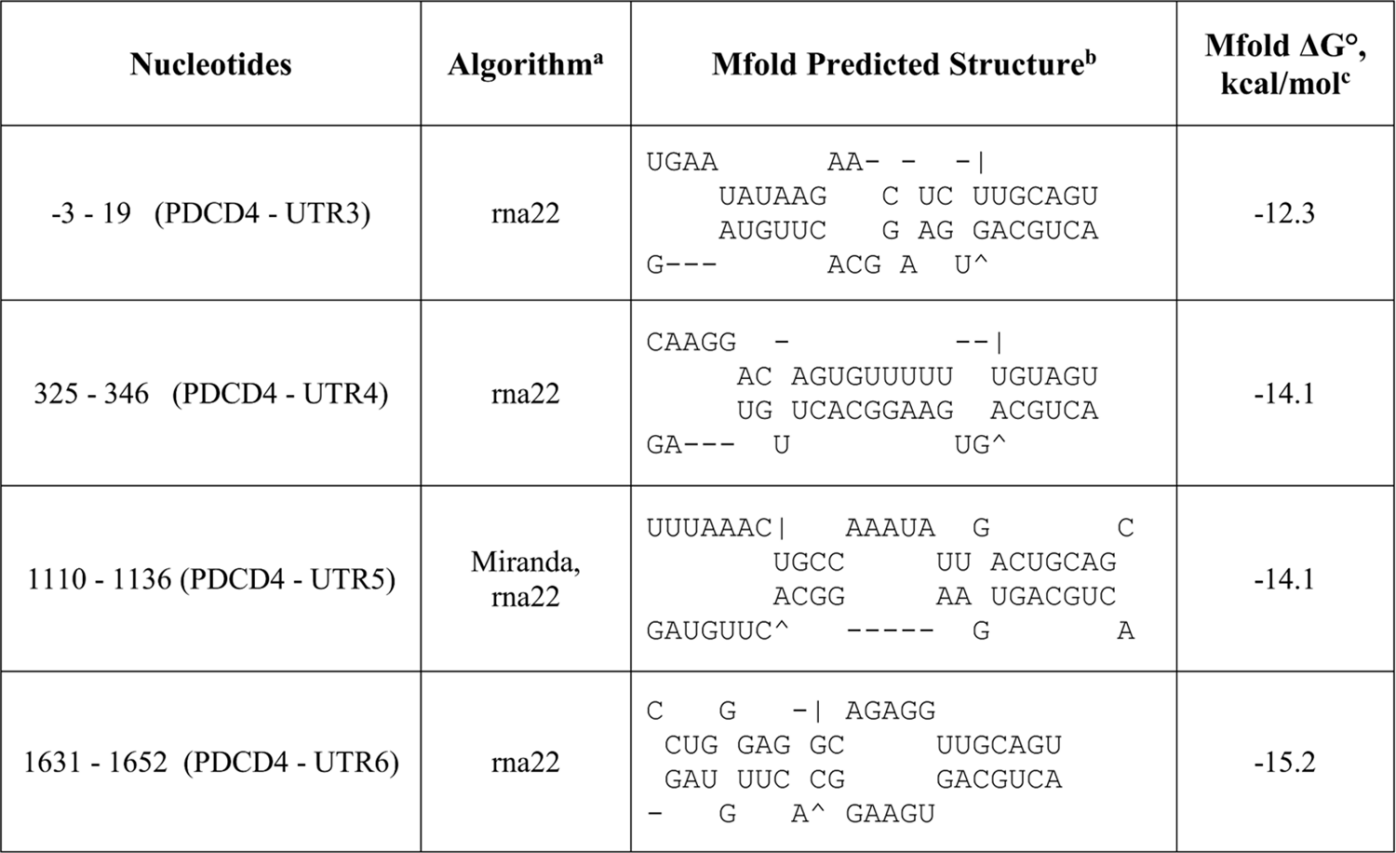
Predicted miR-17-3p passenger strand binding sites in the 3'UTR of *PDCD4* mRNA. ^a^The potential oncomiR:mRNA binding sites were identified by rna22, Targetscan, or miRanda. ^b^Top strand is mRNA (5' ➔ 3') and the bottom strand is oncomiR (3' ➔ 5'). ^c^Calculated at http://mfold.rna.albany.edu/?q=mfold.

**Fig 5:**
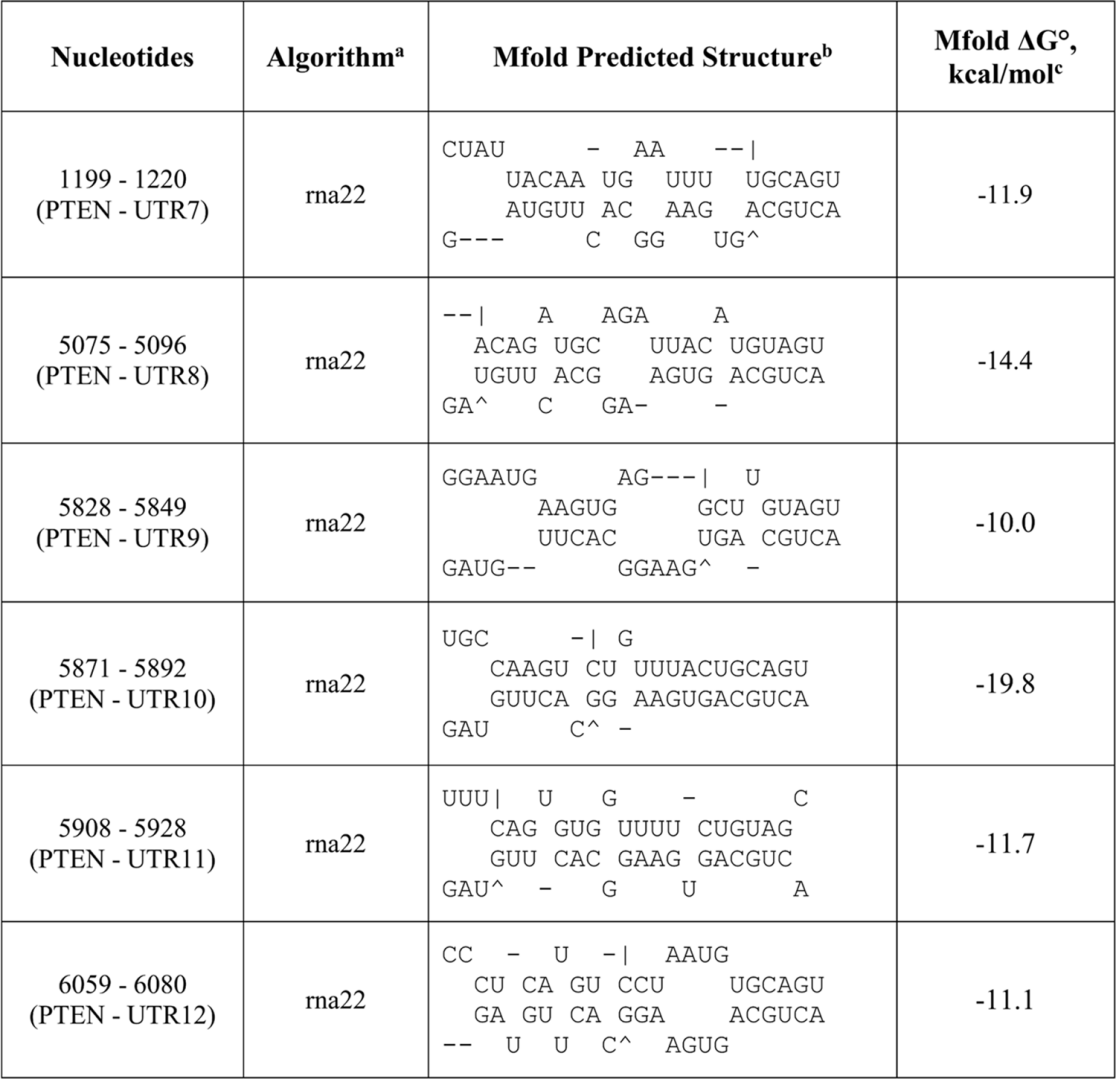
Predicted miR-17-3p passenger strand binding sites in the 3'UTR of *PTEN* mRNA. ^a^The potential oncomiR:mRNA binding sites were identified by rna22, Targetscan, or miRanda. ^b^Top strand is mRNA (5' ➔ 3') and the bottom strand is oncomiR (3' ➔ 5'). ^c^Calculated at http://mfold.rna.albany.edu/?q=mfold.

To test this hypothesis, rna22, TargetScan and miRanda were used to identify potential binding sites for miR-17-3p in the 3’UTR of *PDCD4 and PTEN*. Four potential binding sites for miR-17-3p were noted in the *PDCD4* 3’UTR (Fig 4), while the *PTEN* 3’UTR had six (Fig 5). For each predicted oncomiR:mRNA interaction, the CUGCA motif within the seed region of miR-17-3p is complementary to the corresponding 3’UTR binding sites. This same sequence motif exists in anti-miR-17-5p (Fig 3A). Thus, considering seed pairing as one of the important factors in miRNA:target recognition, all of the miR-17-3p target sites in the 3’UTRs of *PDCD4* and *PTEN* could be putative binding sites for anti-miR-17-5p.

### OncomiR:mRNA duplex structures are stable

Based on the hypothesis that anti-miR-17-5p mimicked miR-17-3p, the interaction between anti-miR-17-5p and miR-17-3p binding sites in the 3’UTR should be stable. We tested our theory by folding anti-miR-17-5p onto miR-17-3p target sites in the *PDCD4* and *PTEN* 3’UTRs. Since Mfold only allows a linear sequence to be the input, we connected the two sequences with a sequence linker (5’-GCGGGGACGC-3’) [30].

**Fig 6:**
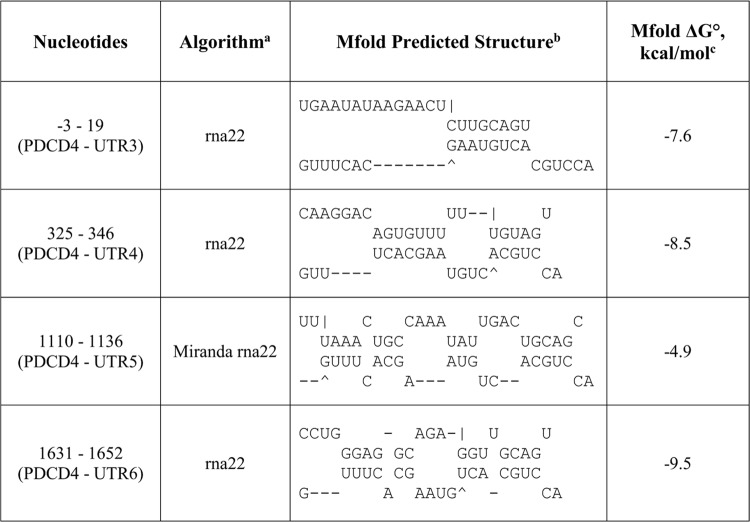
Predicted anti-miR-17-5p binding sites in the 3'UTR of *PDCD4* mRNA as a mimic of miR-17-3p passenger strand. ^a^The potential oncomiR:mRNA binding sites were identified by rna22, Targetscan, or miRanda. ^b^Top strand is mRNA (5' ➔ 3') and the bottom strand is oncomiR (3' ➔ 5'). ^c^Calculated at http://mfold.rna.albany.edu/?q=mfold.

**Fig 7:**
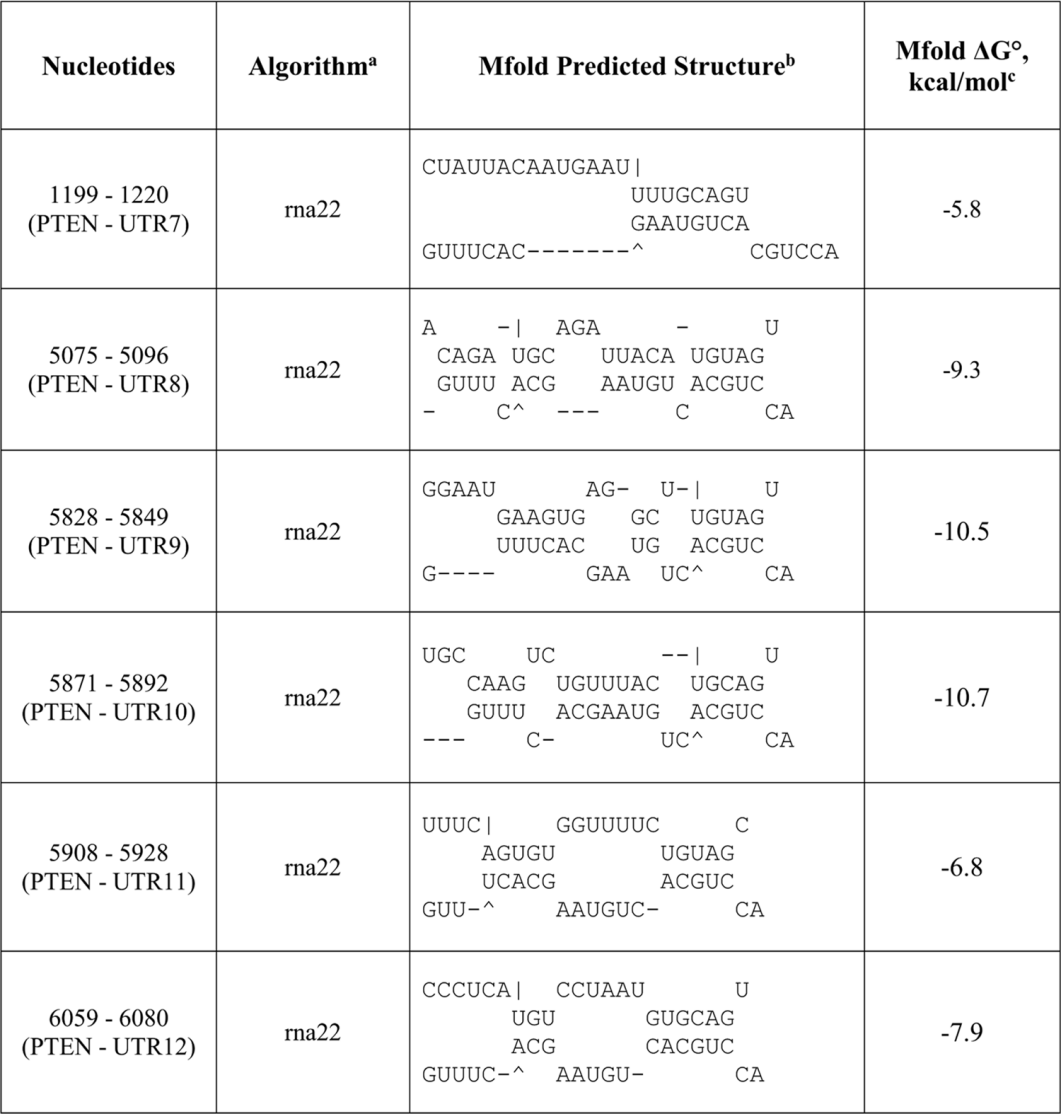
Predicted anti-miR-17-5p binding sites in the 3'UTR of *PTEN* mRNA as a mimic of miR-17-3p passenger strand. ^a^The potential oncomiR:mRNA binding sites were identified by rna22, Targetscan, or miRanda. ^b^Top strand is mRNA (5' ➔ 3') and the bottom strand is oncomiR (3' ➔ 5'). ^c^Calculated at http://mfold.rna.albany.edu/?q=mfold.

**Fig 8:**
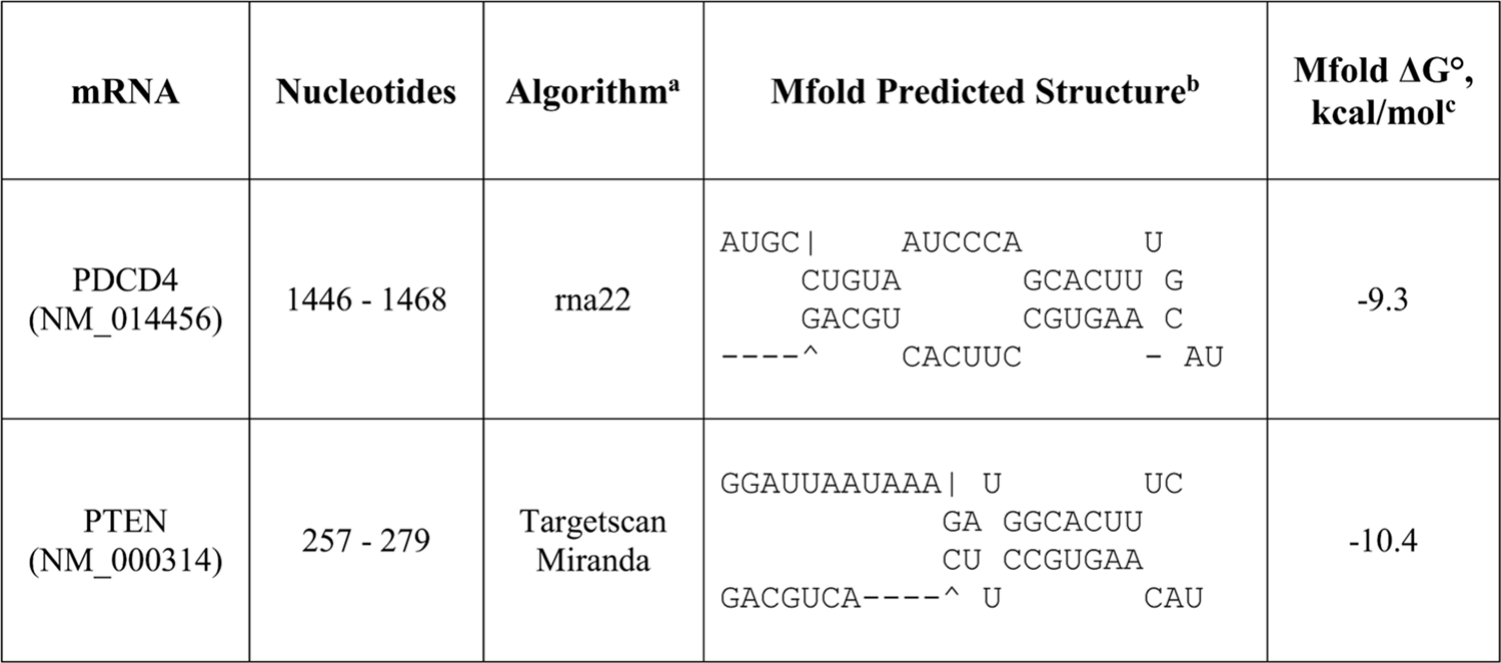
Predicted anti-miR-17-3p binding sites in the 3'UTR of *PDCD4* and *PTEN* mRNAs as a mimic of miR-17-5p passenger strand. ^a^The potential oncomiR:mRNA binding sites were identified by rna22, Targetscan, or miRanda. ^b^Top strand is mRNA (5' ➔ 3') and the bottom strand is oncomiR (3' ➔ 5'). ^c^Calculated at http://mfold.rna.albany.edu/?q=mfold.

As a result, all the anti-miR-17-5p:mRNA pairs were successfully folded with a slightly lower folding energy compared to miR-17-3p:mRNA predictions by rna22 and miRanda (Figs 4–7). However, the lowered folding energy could be compensated by the LNA residues within anti-miR-17-5p, since LNA:RNA duplexes are highly stable [36]. Similarly, anti-miR-17-3p could mimic miR-17-5p and bind to all of the miR-17-5p target sites on the 3’UTR of *PDCD4* and *PTEN* mRNAs (Fig 3A, Fig 8).

### OncomiR:mRNA duplex structures occupy A-form helices

**Fig 9:**
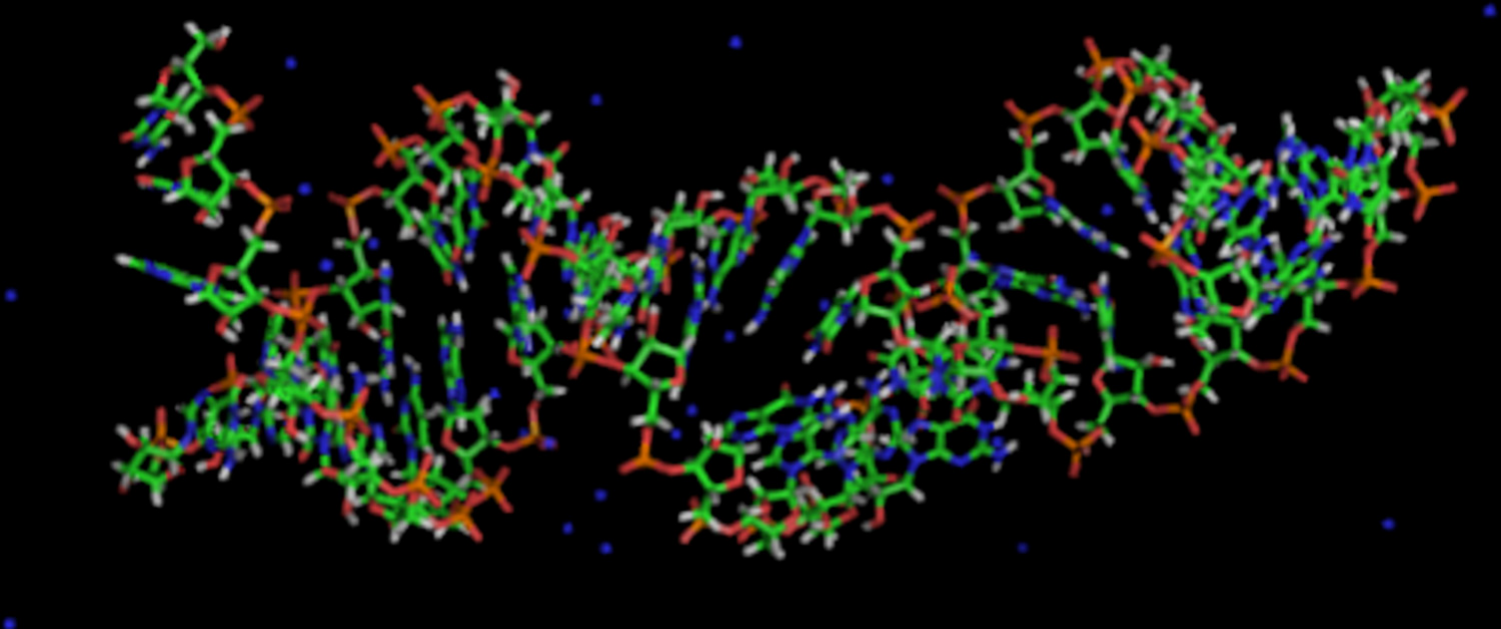
Minimum energy structure predicted with Amber 12 for miR-17-3p:*PTEN* mRNA duplex in explicit H_2_O with 100 mM NaCl, pH 7.0, at 300°K.

Our molecular dynamics calculations predicted stable A-form duplexes for all passenger strand:mRNA targets, as well as for guide strands, despite the mismatches and bulges that appear so distorted in the Mfold presentation. The example of miR-17-3p bound to a *PTEN* 3’UTR site (Fig 9) illustrates the realization that miRNA:mRNA: duplexes could be accommodated in the substrate groove of Ago2, in agreement with an earlier simulation of an 11mer duplex bound to *Thermus thermophilus* Ago [37]. An animated mpg file showing 25 nsec of simulation at 300°K in explicit water with 100 mM NaCl, at pH 7.0, can be viewed in S1 Movie. These results indicated that miRNA inhibitors composed of LNA-DNA could also be bound to Ago when they form duplexes with target miRNAs, since LNA:RNA will pre-organize the helices in A-form.

### Inhibition of the miR-17-3p passenger strand maintained PDCD4 and PTEN protein levels

To determine if the passenger strand was involved in the contradictory results above, we knocked down endogenous miR-17-3p with anti-miR-17-3p and analyzed PDCD4 and PTEN protein expression levels. In contrast to miR-17-5p knockdown (Fig 3C), miR-17-3p knockdown showed no significant changes in PDCD4 or PTEN protein levels (Fig 3D). The maintained protein levels of PDCD4 and PTEN could be a comprehensive outcome of both miR-17-5p and miR-17-3p binding to the *PDCD4* and *PTEN* 3’UTRs. The static result is plausible, because there are more potential binding sites for miR-17-3p on the 3’UTR of *PDCD4* and *PTEN* mRNAs compared to that of miR-17-5p (Fig 4 and Fig 5), although anti-miR-17-3p could act as a miR-17-5p mimic (Fig 3A and Fig 8). In addition, qPCR results showed that the endogenous level of miR-17-5p was about ninety times higher than miR-17-3p in MDA-MB-231 cells (S2A Fig). Thus, knocking down the low level of miR-17-3p passenger strand might not have a noticeable effect.

### miR-21-5p guide strand knockdown elevated PDCD4 protein level

**Fig 10:**
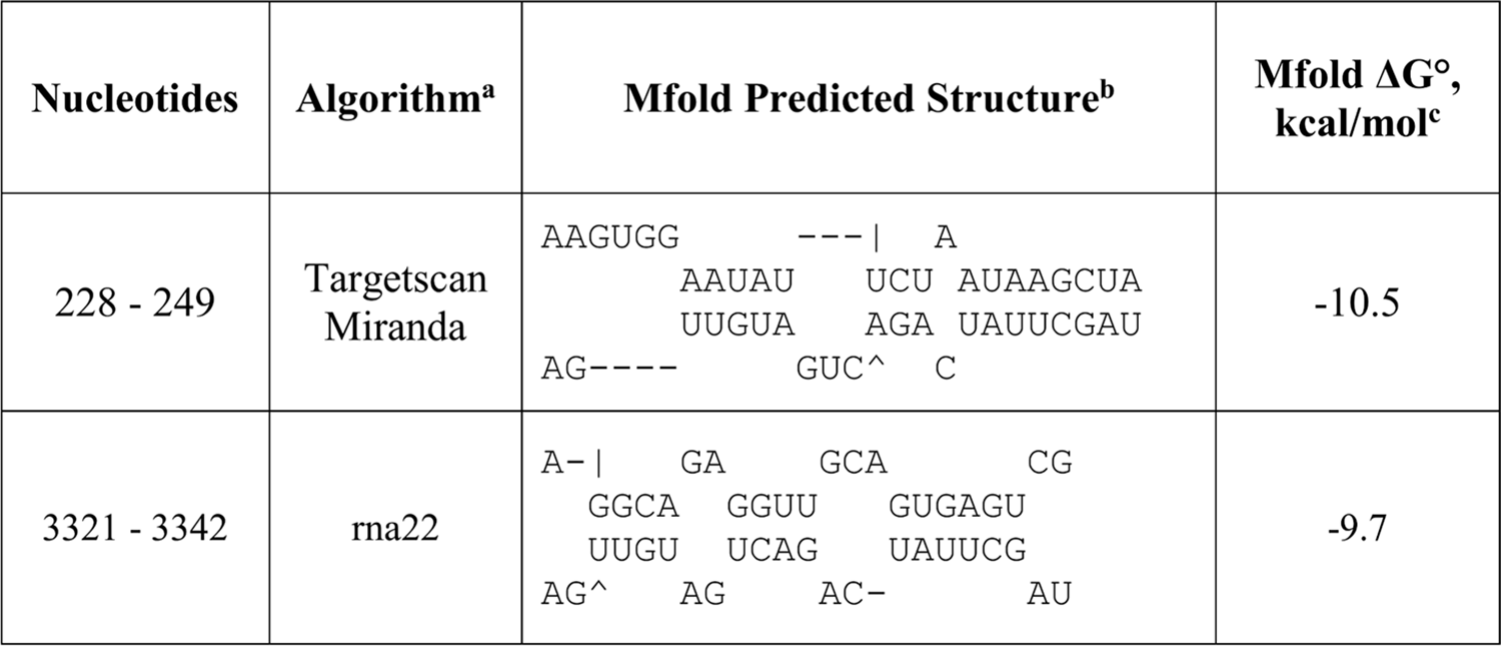
Predicted miR-21-5p guide strand binding sites in the 3'UTR of *PDCD4* mRNA. ^a^The potential oncomiR:mRNA binding sites were identified by rna22, Targetscan, or miRanda. ^b^Top strand is mRNA (5' ➔ 3') and the bottom strand is oncomiR (3' ➔ 5'). ^c^Calculated at http://mfold.rna.albany.edu/?q=mfold.

To further test our hypothesis, we treated MDA-MB-231 cells with anti-miR-21-5p to knock down miR-21-5p, an established regulator of *PDCD4* [38], then measured the effect on PDCD4 protein. rna22, TargetScan, and miRanda predicted that miR-21-5p has two binding sites in the 3’UTR of *PDCD4*, while its passenger strand miR-21-3p has no putative binding sites, unlike miR-17-3p (Fig 10). Anti-miR-21-5p knocked down miR-21-5p by 96±0.15% (Fig 2B), and increased PDCD4 protein expression by 1.4±0.3 fold (Fig 3E). Consistent with the predicted absence of a miR-21-3p site on *PDCD4* mRNA, anti-miR-21-5p did not down-regulate PDCD4 protein level.

### miR-17-3p knockdown increased the steady state level of *PDCD4* mRNA

**Fig 11:**
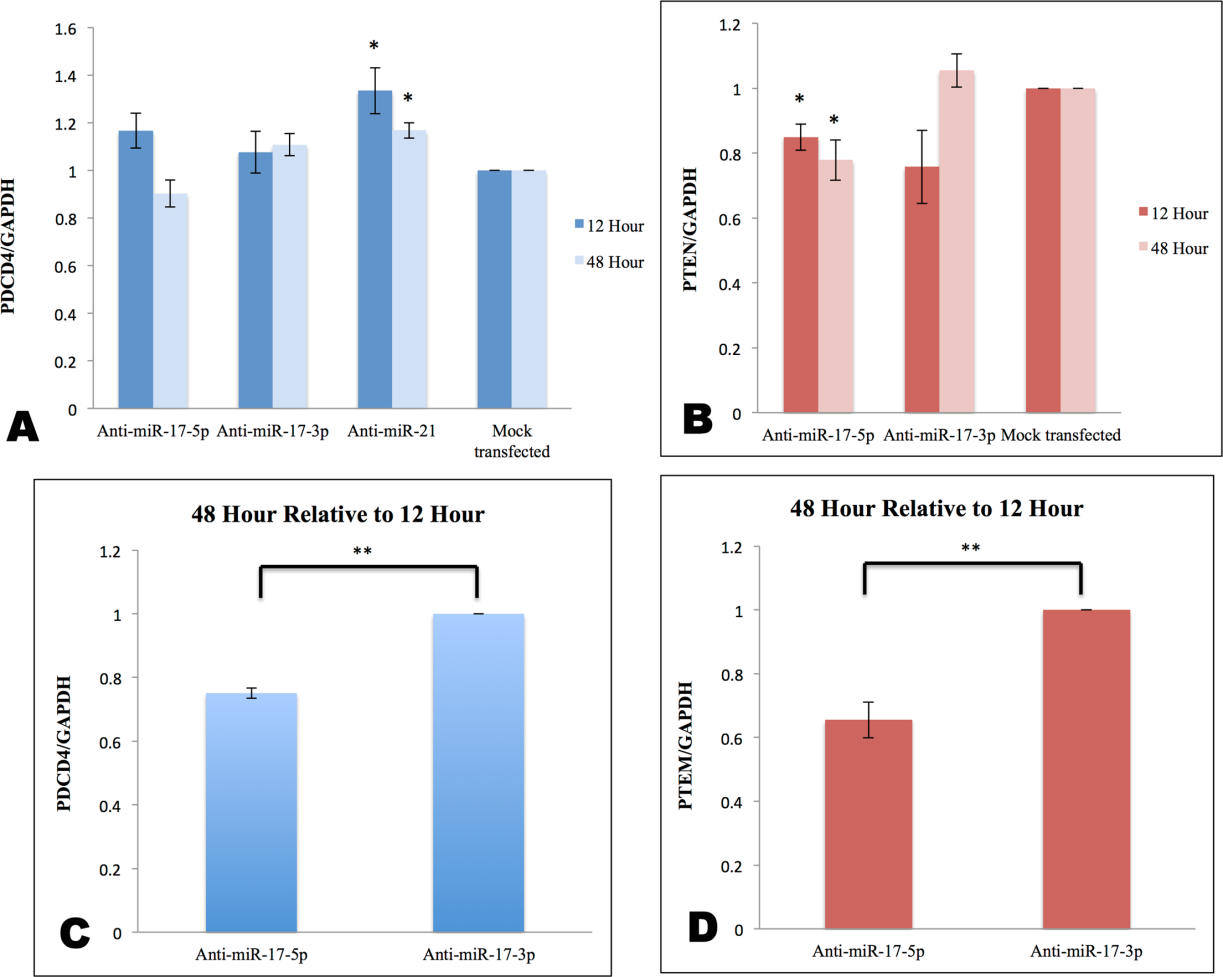
Effect of complementary DNA-LNA chimeras on the *PDCD4* and *PTEN* mRNA levels in MDA-MB-231 cells. **A**: qPCR of *PDCD4* mRNA at 12 hr and 48 hr after transfection. **B**: qPCR of *PTEN* mRNA at 12 hr and 48 hr after transfection. **C**: Relative expression of *PDCD4* mRNA from 12 hr to 48 hr after transfection with anti-miR-17-5p and anti-miR-17-3p. **D**: Relative expression of *PTEN* mRNA from 12 hr to 48 hr after transfection with anti-miR-17-5p and anti-miR-17-3p. Results represent absolute values of miRNA/internal control gene *GAPDH* normalized to mock transfected. Values are the average of three measurements ± s.d. * indicates p<0.05, ** indicates p<0.01.

To investigate whether anti-miR DNA-LNA chimeras influence mRNAs at the transcriptional level, we measured *PDCD4* mRNA levels by qPCR 12 hr and 48 hr post-transfection. At 12 hr and 48 hr, neither miR-17-5p knockdown nor miR-17-3p knockdown correlated with any significant change in *PDCD4* mRNA compared to control (Fig 11A and 11B), while miR-21-5p knockdown significantly increased *PDCD4* mRNA by 33±9.6% at 12 hr and 17±3.3% at 48 hr (Fig 11A). However, *PDCD4* mRNA showed a 25±1.7% increase in its steady state with anti-miR-17-3p relative to anti-miR-17-5p treatment from 12 hr to 48 hr (Fig 11C).

### miR-17-5p knockdown decreased *PTEN* mRNA level, while miR-17-3p knockdown increased the steady state level of *PTEN* mRNA

A similar phenomenon was observed with *PTEN* mRNA. At 12 hr and 48 hr after anti-miR-17-5p transfection, *PTEN* mRNA was significantly decreased by 15±4% at 12 hr and 22±6% at 48 hr, while no significant change was seen with anti-miR-17-3p, compared to control (Fig 11B). Moreover, the steady state of *PTEN* mRNA from 12 hr to 48 hr showed a significant increase (35±6%) with anti-miR-17-3p, compared with anti-miR-17-5p (Fig 11D).

### Both miR-17-5p and miR-17-3p directly affect the translation of *PDCD4* and *PTEN* mRNAs

**Fig 12:**
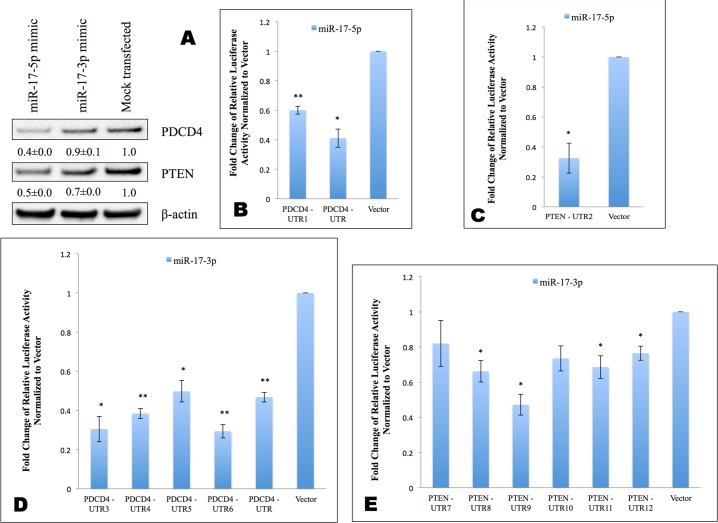
Both miR-17-5p and miR-17-3p can directly modulate the translation of *PDCD4* and *PTEN*. A: PDCD4 and PTEN protein Western blots at 48 hr post transfection with miR-17-5p mimic or miR-17-3p mimic. β-actin was used as loading control. Values are the average of three blots ± s.d. after normalization to β-actin and to control/treatment group. Each blot was subjected to gamma setting adjustments. B—E: Luciferase activity after co-transfecting MDA-MB-231 cells with indicated luciferase reporter constructs in the presence or absence of miRNA mimic. All luciferase signals from pMir-report firefly luciferase vectors are normalized to signals from pRL-TK Renilla luciferase vector. The ratio of normalized signal in the presence of mimic to signal in the absence of mimic for each construct is then calculated. The pMir-report luciferase vector is used as negative control. Results represent fold changes of the above ratio relative to vector control. Values are the average of at least three measurements ± s.e.m * indicates p<0.05, ** indicates p<0.01.

To test the hypothesis that the translation of *PDCD4* and *PTEN* mRNAs are directly inhibited by miR-17-5p and miR-17-3p, we transfected MDA-MB-231 cells with either miR-17-5p mimic or miR-17-3p mimic, and measured the protein levels of PDCD4 and PTEN 48 hr post-transfection. Exogenous miR-17-5p mimic lowered both PDCD4 and PTEN protein levels (Fig 12A). Transfection with miR-17-3p mimic lowered PTEN protein level, but not PDCD4 protein level (Fig 12A). This could be explained by the low endogenous expression level of miR-17-3p passenger strand compared to miR-17-5p guide strand (S2A Fig). Indeed, qPCR showed that transfection with miR-17-5p guide strand mimic increased the measured level of miR-17-5p by over 800-fold (S2B Fig), while transfection with miR-17-3p passenger strand mimic only increased its measured level by 100-fold (S2C Fig).

### Both miR-17-5p and miR-17-3p directly interact with the 3’UTR of *PDCD4* and *PTEN* mRNAs

To study the mechanism of post-transcriptional regulation of *PDCD4* and *PTEN* mRNAs by miR-17-5p and miR-17-3p, we cloned individual binding sites for miR-17-5p or miR-17-3p (Fig 1, Fig 3 and Fig 4) in the 3’UTR of *PDCD4* or *PTEN* mRNAs into luciferase reporter vectors right after the luciferase gene. Since *PDCD4* has not been reported to be a target for miR-17-5p or miR-17-3p, we also cloned the whole 3’UTR of *PDCD4* mRNA into the luciferase reporter vector. The resulting luciferase constructs were expected to express luciferase mRNAs containing the miRNA binding sites as their 3’UTR. MDA-MB-231 cells were co-transfected with each luciferase construct, miR-17-5p or miR-17-3p mimic, and a pRL-TK *Renilla* luciferase vector served as a transfection efficiency control. In parallel, the same transfection was also done without the addition of mimic for each luciferase construct to obtain the baseline luciferase signal. The luciferase light units from each construct were first normalized to that of *Renilla* luciferase vector, then further normalized to the corresponding baseline signal without the mimic. The vector containing the luciferase gene, and the restriction sites in the luciferase 3’UTR, was used as a negative control.

Compared to vector control, miR-17-5p mimic lowered the luciferase activity of both predicted *PDCD4* binding sites and the entire *PDCD4* 3’UTR (Fig 12B), suggesting that miR-17-5p can directly target *PDCD4* mRNA. Similarly, miR-17-5p mimic lowered the luciferase activity of the vectors containing the binding sites from the *PTEN* 3’UTR (Fig 12C). Exogenous miR-17-3p mimic lowered luciferase activities from vector constructs harboring each of the four predicted binding sites, as well as the construct containing the whole 3’UTR of *PDCD4* mRNA (Fig 12D), indicating that both miR-17-5p guide strand and miR-17-3p passenger strand could directly target *PDCD4* mRNA. miR-17-3p mimic lowered luciferase activities of four out of six predicted binding sites in the 3’UTR of *PTEN* mRNA (Fig 12E).

### Anti-miR-17-5p DNA-LNA inhibitors act as miR-17-3p mimics, and reduced the translation of *PDCD4* and *PTEN* mRNAs

**Fig 13:**
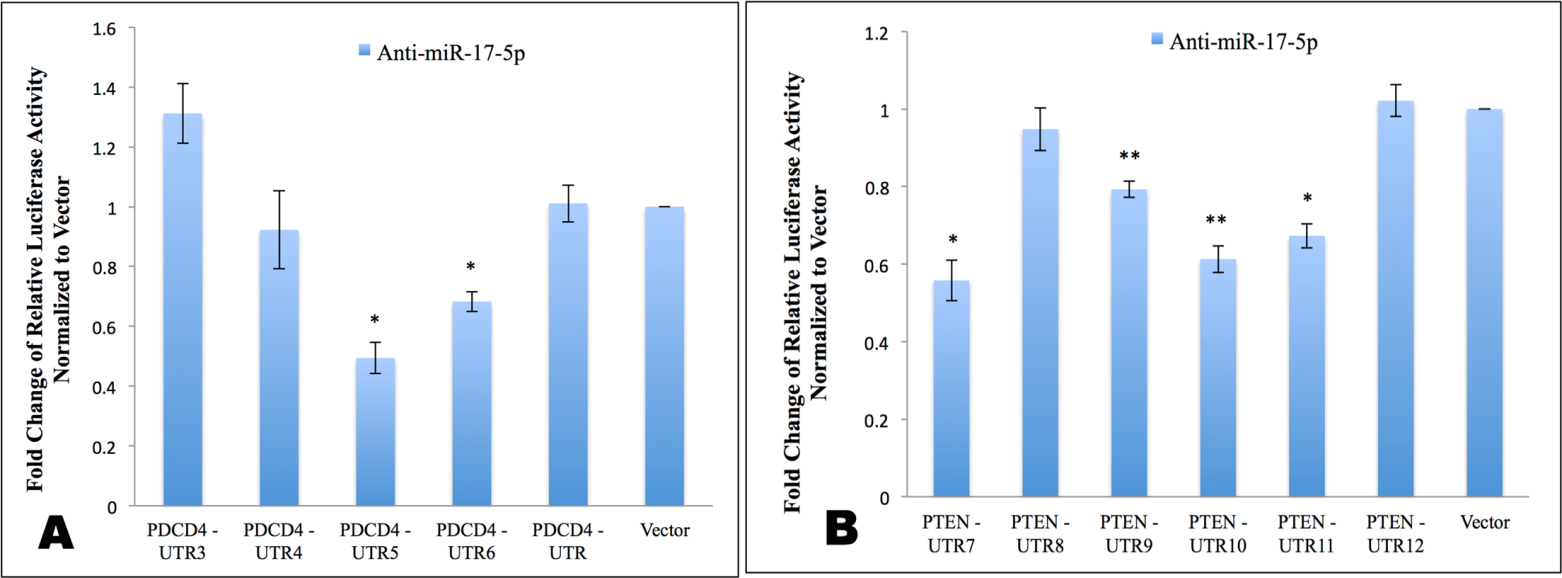
Anti-miR-17-5p DNA-LNA can directly modulate the translation of *PDCD4* and *PTEN* mRNAs through interactions with multiple binding sites from the 3’UTR. Luciferase activity after co-transfecting MDA-MB-231 cells with indicated luciferase reporter constructs in the presence or absence of DNA-LNA inhibitor. A: *PDCD4*. B: *PTEN*. All luciferase signals from pMir-report are normalized to signals from pRL-TK *Renilla* luciferase vector. The ratio of normalized signal in the presence of DNA-LNA inhibitor to signal in the absence of inhibitor for each construct is then calculated. The pMir-report luciferase vector was used as a negative control. Results represent fold changes of the above ratio relative to vector control. Values are the average of at least three measurements ± s.e.m * indicates p<0.05, ** indicates p<0.01.

To investigate whether anti-miR-17-5p DNA-LNA could mimic miR-17-3p by binding to the predicted sites for miR-17-3p in the 3’UTR of *PDCD4* and *PTEN*, we carried out the luciferase experiments by co-transfecting MDA-MB-231 cells with luciferase constructs and anti-miR-17-5p as described above. Compared to vector control, anti-miR-17-5p lowered the activity of luciferase vectors containing two out of four *PDCD4* 3’UTR binding sites predicted for miR-17-3p (Fig 13A). However, it did not affect luciferase activity from the vector containing the entire 3’UTR of *PDCD4* mRNA (Fig 13A). Since the whole *PDCD4* 3’UTR is much longer than the individual binding sites, the secondary and tertiary structure of the entire 3’UTR might be changed after being cloned after the luciferase gene. Anti-miR-17-5p also lowered luciferase activities of constructs harboring four out of six *PTEN* 3’UTR binding sites predicted for miR-17-3p (Fig 13B). These results implied that artificial anti-miR-17-5p DNA-LNA against miR-17-5p guide strand could exert functions of an miRNA by mimicking the passenger strand of miR-17-5p, and could interact with some of the miR-17-3p binding sites in the 3’UTR of *PDCD4* and *PTEN* mRNAs.

## Discussion

In our study, we discovered that in MDA-MB-231 TNBC cells, anti-miR-17-5p DNA-LNA chimera knocked down endogenous miR-17-5p, but surprisingly decreased the protein levels coded by their potential targets *PDCD4* and *PTEN* mRNAs, rather than elevating them. In contrast, anti-miR-17-3p knocked down endogenous miR-17-3p, but maintained PDCD4 and PTEN protein expression (Fig 3C). We ascribe this result to limited binding sites for the miR-17-5p guide strand relative to the miR-17-3p passenger strand.

**Fig 14:**
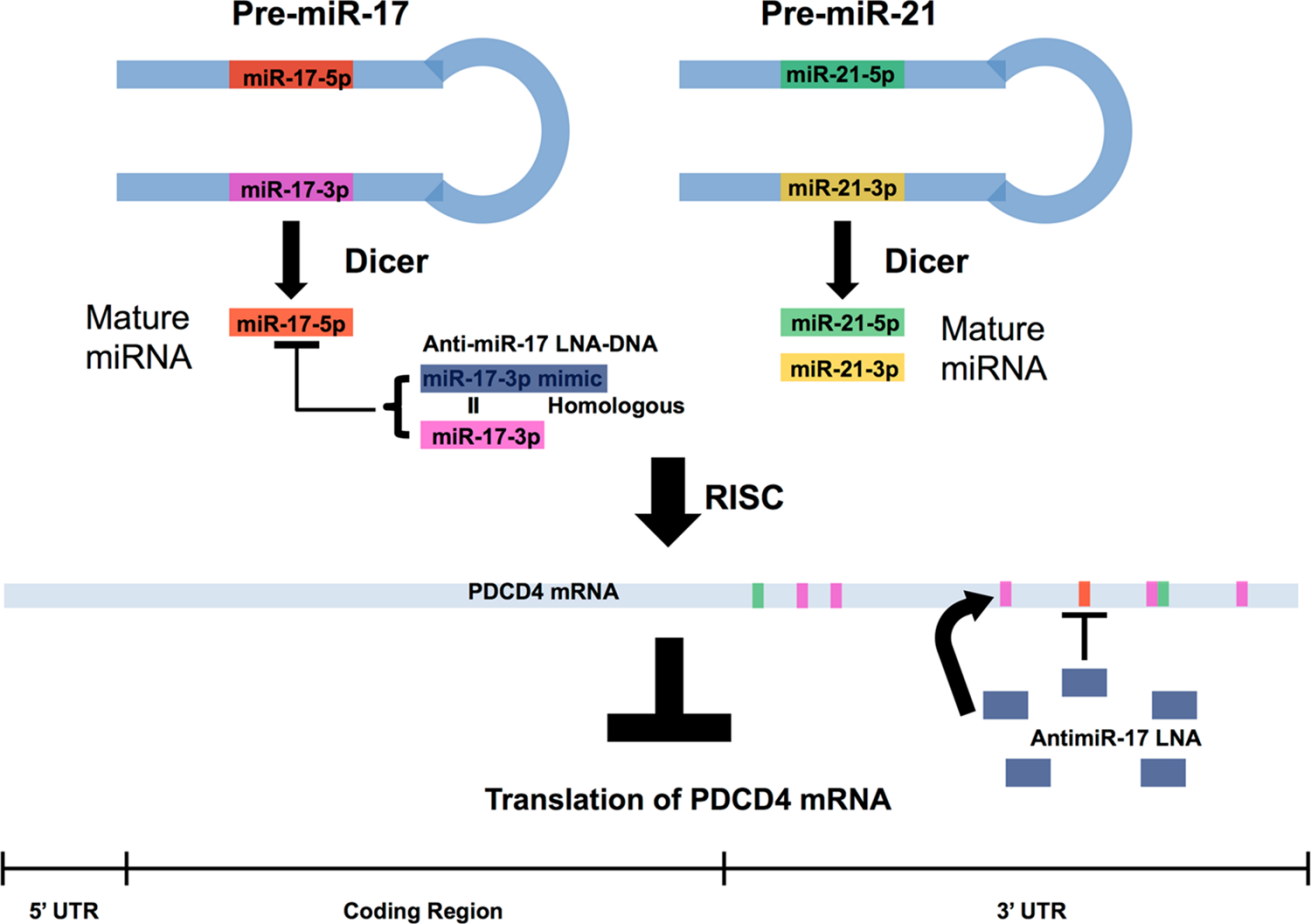
Schematic view of competition between anti-miR-17-5p and miR-17-5p for inhibition of *PDCD4* mRNA.

Through mRNA sequence analysis, we found four putative binding sites for miR-17-3p relative to one for miR-17-5p in the 3’UTR of *PDCD4* mRNA. Thus, while the anti-miR-17-5p could silence miR-17-5p and theoretically alleviate the translational inhibition of *PDCD4* mRNA, as a miR-17-3p mimic it apparently repressed the translation of *PDCD4* mRNA by binding to multiple 3’UTR target sites for miR-17-3p (Fig 13 and Fig 14). Although the endogenous passenger strand miR-17-3p had only modest effects in modulating *PDCD4* and *PTEN* post-transcriptionally (Fig 3D and Fig 12A), the passenger strand mimicking anti-miR-17-5p could target *PDCD4* mRNA more effectively than the miR-17-5p guide strand. This phenomenon could be explained by the excess amount of highly stable anti-miR inhibitors introduced into the cells, which could be associated with Ago, and form strong interactions with the targeting miRNAs. Moreover, the anti-miR inhibitors could be selected by Ago to perform functions of a miRNA due to their pre-organized A-form structure, and could target mRNAs with great side effects through binding to multiple 3’UTR sites.

On the other hand, anti-miR-21-5p did not constitute a passenger strand mimic that competes with the guide strand for binding to target mRNAs. Thus, knocking down miR-21-5p consistently increased PDCD4 protein levels (Fig 3E) and *PDCD4* mRNA levels (Fig 11A) at 12 hr and 48 hr after transfection by relieving translational inhibition of *PDCD4* mRNA by miR-21-5p (Fig 14).

These results suggest that a miRNA inhibitor designed to bind to the mature guide strand could potentially constitute a mimic of the passenger strand. As a result, an oncomiR guide strand inhibitor could fail if passenger strand binding sites exist in the 3’UTR of target mRNAs. This effect was not apparent in an earlier study in lymphocytes that utilized a luciferase vector containing only a fragment of the *PTEN* 3’UTR bearing the single miR-17-5p target [14].

Recent literature has reported some examples of cancer cells with substantial activity exhibited by passenger strand species, including mRNA regulatory activities [39–44]. For example, in hepatocellular carcinoma, miR-17-5p reduced the translation of *PTEN* mRNA, while miR-17-3p directly targeted vimentin mRNA [45]. As a result, miR-17-5p and miR-17-3p cooperatively contribute to the development of hepatocellular carcinoma. The same investigators reported that both the guide strand and the passenger strand of miR-17 targeted sites in *TIMP3* mRNA in prostate cancer cells, thus promoting proliferation and invasion [46].

Those observations in hepatocellular carcinoma and prostate cancer cells, and our own in TNBC cells, contradict the conventional model in which the passenger strands of miRNAs are dissociated and degraded.

Thus, when studying an oncomiR guide strand, one must consider that its passenger strand could also be functional. Furthermore, the expression patterns of passenger strand species vs. guide strand species should also be characterized at various cell cycle stages, since the two oncomiR strands are not always expressed equally as cell growth progresses [47, 48]. Finally, when combining the effect of the two oncomiR strands, different scenarios of consequences must be investigated since the guide strand and the passenger strand miRNAs could theoretically work in synergy, in opposing fashion, or independently. Clearly, oncomiRs regulate genes via a fine-tuning process.

Based on the findings that both oncomiR strands could be functional, precaution must be taken when designing oncomiR knockdown sequences as potential therapeutic or diagnostic agents, since both the guide strand and the passenger strand of an oncomiR might target the same transcript. For research and therapeutic purposes, developing oncomiR inhibitors or mimics that modulate the functionality of either the guide strand or the passenger strand specifically and independently is valuable. Inhibitors specific to one strand or the other will diminish contradictory side effects associated with mimicking the passenger strand.

For future investigations, the activity of passenger strand species from other oncomiRs should also be explored in multiple TNBC cell models. In this study, only two genes were found to be regulated by miR-17 passenger strand. As each oncomiR could target hundreds of gene transcripts, one could expect a comparable number of mRNA targets for oncomiR passenger strand relative to their guide strand.

To characterize the functionality of oncomiR passenger strand species, a global RNA expression analysis will be essential in combination with oncomiR target prediction algorithms and molecular dynamics calculations of complex stability. Gene expression profiling in concordance with oncomiR guide and passenger strand expression will provide us with more powerful insight into the purpose of active passenger strand species.

## Supporting Information

S1 Fig Sequences of primers used to generate luciferase constructs containing individual binding sites for miR-17-5p or miR-17-3p from the 3’UTR of *PDCD4* or *PTEN* mRNAs. (DOCX)

S2 Fig qPCR measurements of miR-17-5p and miR-17-3p in MDA-MB-231 TNBC cells treated with exogenous miR-17-5p mimic or miR-17-3p mimic. Results represent absolute values of miRNA/internal control U6 normalized to miR-17-3p/U6 (A) or mock transfected (B—C). Values are the average of three measurements ± s.d. **A**: qPCR of miR-17-5p and miR-17-3p in MDA-MB-231 cells without mimic. **B:** qPCR of miR-17-5p 48 hr post-transfection with miR-17-5p mimic. **C:** qPCR of miR-17-3p 48 hr post-transfection with miR-17-3p mimic.
(DOCX)

S1 Movie Animated mpg file showing 25 nsec of simulation with Amber 12 at 300K in explicit H_2_O with 100 mM NaCl, pH 7.0, predicted for miR-17-3p:*PTEN* mRNA duplex in 100 mM NaCl, pH 7.0, at 300°K. (MPG)

## Abbreviations

LNA: locked nucleic acid
miRNA: micro ribonucleic acid
miR-17-5p: guide strand of miR-17
miR-17-3p: passenger strand of miR-17
oncomiR: oncogenic miRNA
PBS: phosphate buffered saline
PDCD4: programmed cell death 4
PTEN: phosphatase and tensin homolog
RNA: ribonucleic acid
TNBC: triple negative breast cancer

## References

[1] DeSantis C, Ma J, Bryan L, Jemal A. Breast cancer statistics, 2013. CA Cancer J Clin 2014. 2013;64(1):52–62. 10.3322/caac.21203 24114568

[2] Livasy CA, Karaca G, Nanda R, Tretiakova MS, Olopade OI, Moore DT, et al Phenotypic evaluation of the basal-like subtype of invasive breast carcinoma. Mod Pathol. 2006;19(2):264–71. Epub 2005/12/13. 10.1038/modpathol.3800528 . 16341146

[3] Anders CK, Carey LA. Biology, metastatic patterns, and treatment of patients with triple-negative breast cancer. Clin Breast Cancer. 2009;9 Suppl 2:S73–81. 10.3816/CBC.2009.s.008 19596646 PMC2919761

[4] Calin GA, Croce CM. MicroRNA signatures in human cancers. Nature reviews Cancer. 2006;6(11):857–66. Epub 2006/10/25. 10.1038/nrc1997 . 17060945

[5] Bartel DP. MicroRNAs: genomics, biogenesis, mechanism, and function. Cell. 2004;116(2):281–97. . 14744438 10.1016/s0092-8674(04)00045-5

[6] Meister G, Landthaler M, Patkaniowska A, Dorsett Y, Teng G, Tuschl T. Human Argonaute2 mediates RNA cleavage targeted by miRNAs and siRNAs. Mol Cell. 2004;15(2):185–97. 10.1016/j.molcel.2004.07.007 . 15260970

[7] Lee Y, Ahn C, Han J, Choi H, Kim J, Yim J, et al The nuclear RNase III Drosha initiates microRNA processing. Nature. 2003;425(6956):415–9. . 14508493 10.1038/nature01957

[8] Khvorova A, Reynolds A, Jayasena SD. Functional siRNAs and miRNAs exhibit strand bias. Cell. 2003;115(2):209–16. . 14567918 10.1016/s0092-8674(03)00801-8

[9] Bartel DP. MicroRNAs: target recognition and regulatory functions. Cell. 2009;136(2):215–33. Epub 2009/01/27. 10.1016/j.cell.2009.01.002 . 19167326 PMC3794896

[10] Garzon R, Marcucci G, Croce CM. Targeting microRNAs in cancer: rationale, strategies and challenges. Nat Rev Drug Discov. 2010;9(10):775–89. Epub 2010/10/05. 10.1038/nrd3179 . 20885409 PMC3904431

[11] Volinia S, Calin GA, Liu CG, Ambs S, Cimmino A, Petrocca F, et al A microRNA expression signature of human solid tumors defines cancer gene targets. Proc Natl Acad Sci U S A. 2006;103(7):2257–61. Epub 2006/02/08. 10.1073/pnas.0510565103 16461460 PMC1413718

[12] Petrocca F, Visone R, Onelli MR, Shah MH, Nicoloso MS, de Martino I, et al E2F1-regulated microRNAs impair TGFbeta-dependent cell-cycle arrest and apoptosis in gastric cancer. Cancer Cell. 2008;13(3):272–86. Epub 2008/03/11. 10.1016/j.ccr.2008.02.013 . 18328430

[13] Jin L, Lim M, Zhao S, Sano Y, Simone BA, Savage JE, et al The metastatic potential of triple-negative breast cancer is decreased via caloric restriction-mediated reduction of the miR-17~92 cluster. Breast Cancer Res Treat. 2014;146(1):41–50. Epub 2014/05/28. 10.1007/s10549-014-2978-7 . 24863696 PMC4157915

[14] Xiao C, Srinivasan L, Calado DP, Patterson HC, Zhang B, Wang J, et al Lymphoproliferative disease and autoimmunity in mice with increased miR-17-92 expression in lymphocytes. Nature immunology. 2008;9(4):405–14. Epub 2008/03/11. 10.1038/ni1575 18327259 PMC2533767

[15] Iqbal J, Thike AA, Cheok PY, Tse GM, Tan PH. Insulin growth factor receptor-1 expression and loss of PTEN protein predict early recurrence in triple-negative breast cancer. Histopathology. 2012;61(4):652–9. 10.1111/j.1365-2559.2012.04255.x . 22759273

[16] Wu Y, Sarkissyan M, Elshimali Y, Vadgama JV. Triple negative breast tumors in African-American and Hispanic/Latina women are high in CD44+, low in CD24+, and have loss of PTEN. PLoS One. 2013;8(10):e78259 10.1371/journal.pone.0078259 24167614 PMC3805609

[17] Dong G, Liang X, Wang D, Gao H, Wang L, Wang L, et al High expression of miR-21 in triple-negative breast cancers was correlated with a poor prognosis and promoted tumor cell in vitro proliferation. Medical Oncology. 2014;31(7):1–10. 10.1007/s12032-014-0057-x . 24930006

[18] Frankel LB, Christoffersen NR, Jacobsen A, Lindow M, Krogh A, Lund AH. Programmed cell death 4 (PDCD4) is an important functional target of the microRNA miR-21 in breast cancer cells. J Biol Chem. 2008;283(2):1026–33. 10.1074/jbc.M707224200 . 17991735

[19] Wang D, Huang J, Hu Z. RNA Helicase DDX5 regulates microRNA expression and contributes to cytoskeletal reorganization in basal breast cancer cells. Molecular & Cellular Proteomics. 2012;11(2). 10.1074/mcp.M111.011932 22086602 PMC3277758

[20] Li H, Bian C, Liao L, Li J, Zhao RC. miR-17-5p promotes human breast cancer cell migration and invasion through suppression of HBP1. Breast Cancer Res Treat. 2011;126(3):565–75. Epub 2010/05/28. 10.1007/s10549-010-0954-4 . 20505989

[21] Perou CM, Sorlie T, Eisen MB, van de Rijn M, Jeffrey SS, Rees CA, et al Molecular portraits of human breast tumours. Nature. 2000;406(6797):747–52. 10.1038/35021093 . 10963602

[22] Sorlie T, Perou CM, Tibshirani R, Aas T, Geisler S, Johnsen H, et al Gene expression patterns of breast carcinomas distinguish tumor subclasses with clinical implications. Proc Natl Acad Sci U S A. 2001;98(19):10869–74. 10.1073/pnas.191367098 11553815 PMC58566

[23] Loher P, Rigoutsos I. Interactive exploration of RNA22 microRNA target predictions. Bioinformatics. 2012;28(24):3322–3. Epub 2012/10/18. doi: bts615 [pii]. 10.1093/bioinformatics/bts615 . 23074262

[24] Lewis BP, Burge CB, Bartel DP. Conserved seed pairing, often flanked by adenosines, indicates that thousands of human genes are microRNA targets. Cell. 2005;120(1):15–20. Epub 2005/01/18. doi: S0092867404012607 [pii]. 10.1016/j.cell.2004.12.035 . 15652477

[25] Enright AJ, John B, Gaul U, Tuschl T, Sander C, Marks DS. MicroRNA targets in Drosophila. Genome biology. 2003;5(1):R1 . 14709173 10.1186/gb-2003-5-1-r1 PMC395733

[26] John B, Enright AJ, Aravin A, Tuschl T, Sander C, Marks DS. Human MicroRNA targets. PLoS biology. 2004;2(11):e363. doi: papers2://publication/doi/10.1371/journal.pbio.0020363. 15502875 10.1371/journal.pbio.0020363 PMC521178

[27] Miranda KC, Huynh T, Tay Y, Ang YS, Tam WL, Thomson AM, et al A pattern-based method for the identification of MicroRNA binding sites and their corresponding heteroduplexes. Cell. 2006;126(6):1203–17. doi: papers2://publication/uuid/02756847-46B1-48E3-866C-3E06DAA6806E. 16990141 10.1016/j.cell.2006.07.031

[28] Neve RM, Chin K, Fridlyand J, Yeh J, Baehner FL, Fevr T, et al A collection of breast cancer cell lines for the study of functionally distinct cancer subtypes. Cancer Cell. 2006;10(6):515–27. . 17157791 10.1016/j.ccr.2006.10.008 PMC2730521

[29] Zuker M. Mfold web server for nucleic acid folding and hybridization prediction. Nucleic acids research. 2003;31(13):3406–15. Epub 2003/06/26. 12824337 10.1093/nar/gkg595 PMC169194

[30] Stark A, Brennecke J, Russell RB, Cohen SM. Identification of Drosophila MicroRNA Targets. PLoS Biol. 2003;1(3):E60 . 14691535 10.1371/journal.pbio.0000060 PMC270017

[31] Case DA, Darden TA, Cheatham TE III, Simmerling CL, Wang J, Duke RE, et al. Amber 12. San Francisco: University of California; 2012.

[32] Duan Y, Wu C, Chowdhury S, Lee MC, Xiong G, Zhang W, et al A point-charge force field for molecular mechanics simulations of proteins based on condensed-phase quantum mechanical calculations. J Comput Chem. 2003;24(16):1999–2012. Epub 2003/10/08. 10.1002/jcc.10349 . 14531054

[33] Sanders JM, Wampole ME, Chen C-P, Sethi D, Singh A, Dupradeau FY, et al Effects of hypoxanthine substitution in peptide nucleic acids targeting *KRAS2* oncogenic mRNA molecules: theory and experiment. Journal of Physical Chemistry B. 2013;117(39):11584–95. 10.1021/jp4064966 . 23972113 PMC3946533

[34] Sonar MV, Wampole ME, Jin Y-Y, Chen C-P, Thakur ML, Wickstrom E. Fluorescence detection of *KRAS2* mRNA hybridization in lung cancer cells with PNA-peptides containing an internal thiazole orange. Bioconjugate Chemistry. 2014;25: in press. 10.1021/bc500304m . 25180641 PMC4166030

[35] Livak KJ, Schmittgen TD. Analysis of relative gene expression data using real-time quantitative PCR and the 2(-Delta Delta C(T)) Method. Methods. 2001;25(4):402–8. 10.1006/meth.2001.1262 . 11846609

[36] Vester B, Wengel J. LNA (locked nucleic acid): high-affinity targeting of complementary RNA and DNA. Biochemistry. 2004;43(42):13233–41. Epub 2004/10/20. 10.1021/bi0485732 . 15491130

[37] Xia Z, Clark P, Huynh T, Loher P, Zhao Y, Chen HW, et al Molecular dynamics simulations of Ago silencing complexes reveal a large repertoire of admissible 'seed-less' targets. Sci Rep. 2012;2:569 Epub 2012/08/14. 10.1038/srep00569 22888400 PMC3415692

[38] Lu Z, Liu M, Stribinskis V, Klinge CM, Ramos KS, Colburn NH, et al MicroRNA-21 promotes cell transformation by targeting the programmed cell death 4 gene. Oncogene. 2008;27(31):4373–9. Epub 2008/04/01. doi: onc200872 [pii]. 10.1038/onc.2008.72 . 18372920 PMC11968769

[39] Ro S, Park C, Young D, Sanders KM, Yan W. Tissue-dependent paired expression of miRNAs. Nucleic Acids Res. 2007;35(17):5944–53. Epub 2007/08/30. 10.1093/nar/gkm641 17726050 PMC2034466

[40] Yang JS, Phillips MD, Betel D, Mu P, Ventura A, Siepel AC, et al Widespread regulatory activity of vertebrate microRNA* species. Rna. 2011;17(2):312–26. Epub 2010/12/24. 10.1261/rna.2537911 21177881 PMC3022280

[41] Okamura K, Phillips MD, Tyler DM, Duan H, Chou YT, Lai EC. The regulatory activity of microRNA* species has substantial influence on microRNA and 3' UTR evolution. Nature structural & molecular biology. 2008;15(4):354–63. Epub 2008/04/01. 10.1038/nsmb.1409 18376413 PMC2698667

[42] Lee DY, Shatseva T, Jeyapalan Z, Du WW, Deng Z, Yang BB. A 3'-untranslated region (3'UTR) induces organ adhesion by regulating miR-199a* functions. PloS one. 2009;4(2):e4527 Epub 2009/02/19. 10.1371/journal.pone.0004527 19223980 PMC2638016

[43] Guo L, Liang T, Gu W, Xu Y, Bai Y, Lu Z. Cross-mapping events in miRNAs reveal potential miRNA-mimics and evolutionary implications. PLoS One. 2011;6(5):e20517 Epub 2011/06/04. 10.1371/journal.pone.0020517 21637827 PMC3102724

[44] Guennewig B, Roos M, Dogar AM, Gebert LF, Zagalak JA, Vongrad V, et al Synthetic pre-microRNAs reveal dual-strand activity of miR-34a on TNF-alpha. RNA. 2014;20(1):61–75. 10.1261/rna.038968.113 24249224 PMC3866645

[45] Shan SW, Fang L, Shatseva T, Rutnam ZJ, Yang X, Du W, et al Mature miR-17-5p and passenger miR-17-3p induce hepatocellular carcinoma by targeting PTEN, GalNT7 and vimentin in different signal pathways. J Cell Sci. 2013;126(Pt 6):1517–30. Epub 2013/02/19. doi: jcs.122895 [pii]. 10.1242/jcs.122895 . 23418359

[46] Yang X, Du WW, Li H, Liu F, Khorshidi A, Rutnam ZJ, et al Both mature miR-17-5p and passenger strand miR-17-3p target TIMP3 and induce prostate tumor growth and invasion. Nucleic Acids Res. 2013. Epub 2013/08/31. doi: gkt680 [pii]. 10.1093/nar/gkt680 . 23990326 PMC3834805

[47] Wang Q, Li YC, Wang J, Kong J, Qi Y, Quigg RJ, et al miR-17-92 cluster accelerates adipocyte differentiation by negatively regulating tumor-suppressor Rb2/p130. Proc Natl Acad Sci U S A. 2008;105(8):2889–94. 10.1073/pnas.0800178105 18287052 PMC2268555

[48] Shan SW, Lee DY, Deng Z, Shatseva T, Jeyapalan Z, Du WW, et al MicroRNA MiR-17 retards tissue growth and represses fibronectin expression. Nat Cell Biol. 2009;11(8):1031–8. 10.1038/ncb1917 . 19633662

