# Supplementary material for: Non-Specific Blocking of miR-17-5p Guide Strand in Triple Negative Breast Cancer Cells by Amplifying Passenger Strand Activity
Source: PLoS One. 2015 Dec 2;10(12):e0142574. doi: 10.1371/journal.pone.0142574 (PMC4667903; doi:10.1371/journal.pone.0142574)
Supplement: S1 Fig — (DOCX) [file pone.0142574.s001.docx]

**S1 Fig.** **Sequences of primers used to generate luciferase constructs containing individual binding sites for miR-17-5p or miR-17-3p from the 3’UTR of *PDCD4* or *PTEN* mRNAs.**

**Primers harboring miR-17-5p binding sites in the 3’UTR of *PDCD4* mRNA**

**PDCD4 – UTR1 Sense**

CTAGTTGGGCACGGTGGCTCATGCCTGTAATCCCAGCACTTTGGGAGGCCGAGGTGGGA

**PDCD4 – UTR1 Antisense**

AGCTTCCCACCTCGGCCTCCCAAAGTGCTGGGATTACAGGCATGAGCCACCGTGCCCAA

**Primers harboring miR-17-5p binding sites in the 3’UTR of *PTEN* mRNA**

**PTEN – UTR2 Sense**

CTAGTTTTAAAATTCAATTAGGATTAATAAAGATGGCACTTTCCCGTTTTATTCCAGTA

**PTEN – UTR2 Antisense**

AGCTTTTTATAACATCTAAGACTGCAAGAGTTCTTATATTCAGTAGCTCTCTGGTTTA

**Oligomers harboring miR-17-3p binding sites in the 3’UTR of *PDCD4* mRNA**

**PDCD4 – UTR3 Sense**

CTAGTAAACCAGAGAGCTACTGAATATAAGAACTCTTGCAGTCTTAGATGTTATAAAA

**PDCD4 – UTR3 Antisense**

AGCTTTTTATAACATCTAAGACTGCAAGAGTTCTTATATTCAGTAGCTCTCTGGTTTA

**PDCD4 – UTR4 Sense**

CTAGTGCCACTCCTTTCTTTCAAGGACAGTGTTTTTTGTAGTAAAATCACTGGTTTAA

**PDCD4 – UTR4 Antisense**

AGCTTTAAACCAGTGATTTTACTACAAAAAACACTGTCCTTGAAAGAAAGGAGTGGCA

**PDCD4 – UTR5 Sense**

CTAGTACGTCTGTGCTAATTTAAACTGCCAAATATTGACTGCAGCAAACAAGAATTATA

**PDCD4 – UTR5 Antisense**

AGCTTATAATTCTTGTTTGCTGCAGTCAATATTTGGCAGTTTAAATTAGCACAGACGTA

**PDCD4 – UTR6 Sense**

CTAGTGGAGAATTGCTTGAACCTGGGAGGCAGAGGTTGCAGTGAGTCGAGATGGTGCA

**PDCD4 – UTR6 Antisense**

AGCTTGCACCATCTCGACTCACTGCAACCTCTGCCTCCCAGGTTCAAGCAATTCTCCA

**Primers harboring miR-17-3p binding sites in the 3’UTR of *PTEN* mRNA**

**PTEN – UTR7 Sense**

CTAGTTTGACCTTACACATTCTATTACAATGAATTTTGCAGTTTTGCACATTTTTTAA

**PTEN – UTR7 Antisense**

AGCTTTAAAAAATGTGCAAAACTGCAAAATTCATTGTAATAGAATGTGTAAGGTCAAA

**PTEN – UTR8 Sense**

CTAGTTTACTTTCTAATGCCACAGATGCAGATTACATGTAGTTATTGAGAATCCTTTA

**PTEN – UTR8 Antisense**

AGCTTAAAGGATTCTCAATAACTACATGTAATCTGCATCTGTGGCATTAGAAAGTAAA

**PTEN – UTR9 Sense**

CTAGTAGATTTTATTTGTGTGGAATGAAGTGAGGCTTGTAGTCATGGTTCTAGTGTTA

**PTEN – UTR9 Antisense**

AGCTTAACACTAGAACCATGACTACAAGCCTCACTTCATTCCACACAAATAAAATCTA

**PTEN – UTR10 Sense**

CTAGTTCTAGTGTTTCAGTTTGCCAAGTCTGTTTACTGCAGTGAAATTCATCAAATGA

**PTEN – UTR10 Antisense**

AGCTTCATTTGATGAATTTCACTGCAGTAAACAGACTTGGCAAACTGAAACACTAGAA

**PTEN – UTR11 Sense**

CTAGTGAAATTCATCAAATGTTTCAGTGTGGTTTTCTGTAGCCTATCATTTACTGGA

**PTEN – UTR11 Antisense**

AGCTTCCAGTAAATGATAGGCTACAGAAAACCACACTGAAACATTTGATGAATTTCA

**PTEN – UTR12 Sense**

CTAGTTTTTCCATTAAATTGCCCTCATGTCCTAATGTGCAGTTTGTAAGTGTGTGTGA

**PTEN – UTR12 Antisense**

AGCTTCACACACACTTACAAACTGCACATTAGGACATGAGGGCAATTTAATGGAAAAA
