# Supplementary material for: Non-Specific Blocking of miR-17-5p Guide Strand in Triple Negative Breast Cancer Cells by Amplifying Passenger Strand Activity
Source: PLoS One. 2015 Dec 2;10(12):e0142574. doi: 10.1371/journal.pone.0142574 (PMC4667903; doi:10.1371/journal.pone.0142574)
Supplement: S2 Fig — Results represent absolute values of miRNA/internal control U6 normalized to miR-17-3p/U6 (A) or mock transfected (B—C). Values are the average of three measurements ± s.d. A: qPCR of miR-17-5p and miR-17-3p in MDA-MB-231 cells without mimic. B: qPCR of miR-17-5p 48 hr post-transfection with miR-17-5p mimic. C: qPCR of miR-17-3p 48 hr post-transfection with miR-17-3p mimic. (DOCX) [file pone.0142574.s002.docx]

**S2 Fig. qPCR measurements of miR-17-5p and miR-17-3p in MDA-MB-231 TNBC cells treated with exogenous miR-17-5p mimic or miR-17-3p mimic.** Results represent absolute values of miRNA/internal control U6 normalized to miR-17-3p/U6 (A) or mock transfected (B - C). Values are the average of three measurements ± s.d.

**S2A**

**S2A**: qPCR of miR-17-5p and miR-17-3p in MDA-MB-231 cells without mimic.

S**2B**

**S2B:** qPCR of miR-17-5p 48 hr post-transfection with miR-17-5p mimic.

S**2C**

**S2C:** qPCR of miR-17-3p 48 hr post-transfection with miR-17-3p mimic.
